# Posttraumatic growth in parents long after their child’s death from cancer—a cross-sectional survey in Switzerland

**DOI:** 10.1007/s00520-025-09892-x

**Published:** 2025-10-01

**Authors:** Eddy Carolina Pedraza, Peter Francis Raguindin, Anna Katharina Vokinger, Eva De Clercq, Manya Jerina Hendriks, Eva Maria Tinner, André Oscar von Bueren, Katrin Scheinemann, Eva Bergsträsser, Gisela Michel

**Affiliations:** 1 Faculty of Health Sciences and Medicine, University of Lucerne, Lucerne, Switzerland; 2 Inselspital, University Children’s Hospital Bern, Bern, Switzerland; 3 Pediatric Palliative Care, University Children’s Hospital, Zurich, Switzerland; 4 Department of Pediatrics Obstetrics and Gynecology Division of Pediatric Hematologyand, Oncology University Hospital of Geneva, Geneva, Switzerland; 5 CANSEARCH Research Platform for Pediatric Oncology and Hematology, Faculty of Medicine, Department of Pediatrics, Gynecology and Obstetrics, University of Geneva, Geneva, Switzerland; 6 Division of Hematology-Oncology, Children’s Hospital of Eastern Switzerland, St Gallen, Switzerland

**Keywords:** Bereaved parents, Bereavement care, Posttraumatic growth, Positive changes, Childhood cancer

## Abstract

**Background:**

The death of a child is one of the most devastating experiences for parents, yet some may experience positive changes known as posttraumatic growth (PTG). We aimed to describe PTG in bereaved parents whose child died of cancer, compare it to PTG in parents of childhood cancer survivors, and identify sociodemographic and child-related characteristics associated with PTG in bereaved parents.

**Methods:**

This multicenter cross-sectional study included parents who lost a child to cancer (diagnosed ≤ 18 years and > 1 year after death). Data from the Swiss Childhood Cancer Survivor Study-Parents were used for comparison. We used the Posttraumatic Growth Inventory (PTGI) assessing five domains (appreciation of life, new possibilities, personal strength, relating to others, and spiritual change) on a 6-point scale.

**Results:**

We included 103 bereaved parents (mean age = 53.7 years, SD = 8.3; 66.7% female) of 81 deceased children (mean age at death = 9.5 years, SD = 5.9; mean time after death = 11.3 years, range 2–24 years). The PTG sum score was 63.2 (SD = 23.3, range = 4–105), which was higher than in parents of survivors (mean = 51.4; SD = 21.0, *p* = 0.002). Bereaved parents reported higher mean domain scores in *appreciation of life* (3.53, SD = 1.17 vs. 3.01, SD = 1.17, *p* = 0.003), *personal strength* (3.42, SD = 1.25 vs. 2.75, SD = 1.17, *p* = 0.001), *relating to others* (3.09, SD = 1.09 vs. 2.61, SD = 1.04 *p* 0.004), and *new possibilities* (2.69, SD = 1.26 vs. 1.97, SD = 1.17, *p* < 0.001). Parents within 10 years of their child’s death and those practicing religion reported higher PTG.

**Conclusion:**

Our findings reveal that cancer-bereaved parents may experience PTG after their child’s death, embracing unique positive changes according to their circumstances and influencing factors.

**Supplementary Information:**

The online version contains supplementary material available at 10.1007/s00520-025-09892-x.

## Background

The loss of a child is a devastating event for parents. Although a variety of experiences and psychosocial comorbidities, such as anxiety, depression, prolonged grief, and poor quality of life may be part of the trajectory of parental grief [1–3], research has also shown that adverse experiences can lead to self-perceived positive changes known as posttraumatic growth (PTG) [4–6]. Thus, individuals may experience both distress and growth concurrently.

Growth may arise from the various internal and external coping mechanisms a person adopts after experiencing trauma. According to the Posttraumatic Growth Model [5], managing emotional distress, personality traits, social support, and the cognitive processing and restructuring of traumatic experiences influence development of PTG. The “Growth Following Adversity” model [7] highlights that individuals can acquire new perspectives after trauma by reinterpreting the event and its aftermath as part of posttraumatic growth.

Research on PTG in parents who have lost their child to cancer is scarce. The experience of losing a child to cancer is unique and might differ from other types of child loss [8–10]. This may be attributed to the ambivalent feelings and emotions experienced throughout the disease trajectory, e.g., balancing hope for a cure with the fear of death [10–12]. A systematic review on the potential factors that promote posttraumatic growth (PTG) and resilience in bereaved parents emphasized the critical role of support and communication with palliative care teams and other bereaved families. Studies in other populations also showed the importance of coping strategies (continuing bonds and meaning-making), gender, time after death, cultural background, and religion as factors influencing PTG development [13, 14]. While most existing studies described PTG qualitatively, only few have measured and compared PTG quantitatively.

Currently, a limited number of studies focused on the positive changes that may occur over an extended period following the death of a loved one. Most studies in bereaved parents focused on the early stage of grief, until 2 years after a child’s death [15]. A qualitative study among parents 5 years after the death of their child to cancer in comparison with survivors revealed similar percentages of positive experiences among mothers in both groups, while bereaved fathers expressed fewer positive experiences than the comparison group [12]. Despite this, research specifically addressing PTG beyond 5 years after the child’s death remains notably scarce. While many studies focus on the initial years of bereavement [15, 16], other researchers have emphasized that grief can persist beyond this period [17, 18]. Some studies indicated that the intensity of grief experiences may increase during the third year following the child’s death. Furthermore, parents may continue to experience distress and loss, including the phenomenon of an “empty space,” 7 to 9 years later [18]. We aimed to (a) describe the PTG experienced by bereaved parents following the loss of their child to cancer in the long term, (b) compare the PTG of bereaved parents to that of parents whose child survived cancer, and (c) identify sociodemographic and child-related characteristics associated with PTG in bereaved parents.

## Methods

This is a cross-sectional study, part of a larger mixed-method project, conducted from July 2022 to July 2023 in three hospitals in the German-speaking region of Switzerland. We used the Consensus-Based Checklist for Reporting of Survey Studies (CROSS) as a framework in reporting the methods and results (Appendix Table S1) [19].

### Setting and population

#### Recruitment and data collection from bereaved parents

Eligible participants were bereaved parents who had lost a child to cancer: (a) diagnosed with cancer at ≤ 18 years of age, (b) death attributed to cancer, (c) a minimum of 1 year since the child’s death, and (d) childhood cancer treatment in Switzerland.

Participating hospitals received a list of eligible parents from the Swiss Childhood Cancer Registry (ChCR). Hospitals could exclude bereaved parents if they were deemed unsuitable for the study for medical or personal reasons (e.g., parents known to be too vulnerable to participate). Addresses that were provided by the ChCR and the hospital database were cross-referenced and updated, if necessary.

Eligible parents were sent information letters from their former treating clinic including an information letter about the study signed by the head of the clinic and the principal investigator of the study, questionnaires including consent forms (one for each parent), information flyers, and prepaid return envelopes. Reminder phone calls were conducted by two hospitals. One of the participant hospitals chose to forego this step. The earliest calls were made 5 weeks after the questionnaire was sent to parents. Information about the study was also disseminated via cancer and bereavement support organizations. This enabled bereaved parents who were not contacted through the hospital to contact the study team directly. Parents had the possibility of completing the questionnaire online (Qualtrics™, Provo, Utah, US) or on paper. The questionnaire was available in German.

Parents were asked to fill in their own questionnaire individually (mother and father separately). Responses of printed questionnaires were entered manually into the electronic database. To ensure the accuracy of data entry, 23.8% (*N* = 15/63) of the printed questionnaires were entered independently by two study staff members (AKV, KO, LH, ECP—interrater reliability, kappa 0.93).

#### Comparison population: parents of childhood cancer survivors

For the comparison group, we used data from parents of childhood cancer survivors (CCS parents). Data were collected as part of the Swiss Childhood Cancer Survivor Study–Parents (SCCSS-Parents) [20–25]. In short, eligible parents were identified by the ChCR which registers all children diagnosed with cancer in Switzerland. Parents of children who were aged ≤ 16 years (born between 1976 and 2009) and Swiss resident at diagnosis, ≥ 5 years post-diagnosis, and aged ≥ 20 years at the time of the study were eligible [20].

The contact details were updated in the ChCR if necessary and questionnaires were sent by postal mail from the former treatment clinic. The contact period was between January 2017 and February 2018. Questionnaires were made available in French, German, and Italian. In case of non-response, a reminder was sent 4–6 weeks after the first invitation.

### Measurements

We used the Posttraumatic Growth Inventory (PTGI) to measure PTG in parents. This tool underwent content and psychometric validation in Switzerland [26] and had been used in other Swiss studies [20, 27, 28]. PTGI is a 21-item scale with responses from 0 “Did not experience” to 5 “Experienced to a high degree” (minimum score 0, maximum score 105) (Cronbach’s α = 0.94). It examines 5 domains, namely *personal strength* (4 items, sum score 0–20, α = 0.83), *new possibilities* (5 items, 0–25, α = 0.85), *relating to others* (7 items, 0–35, α = 0.86), *spiritual change* (2 items, 0–10, α = 0.93), and *appreciation of life* (3 items, 0–15, α = 0.79). For missing item-response, we imputed the mean of the items within a domain, if a participant had > 50% of the items answered within the domain [29]. The *PTG sum score* was computed by summing up the item responses of each participant. The mean of each domain was also computed.

#### Sociodemographic and child-related characteristics

We collected sociodemographic information of parents in the questionnaire: age (in years), sex (male, female), employment (employed, unemployed/retired), education (compulsory vocational, upper secondary, university), partnership status (single/divorced, married), practicing religion (yes, no), migration status (migration background, non-migration background). Additionally, we collected clinical information related to the deceased child: age at diagnosis (years), age at death (years), sex (male, female), cancer diagnosis (leukemia/lymphoma, CNS tumor, others), location of death (hospital facility, home), and time after death (years).

### Data analysis

All analyses were performed using Stata 18.5 (StataCorp, Texas, USA). We summarized the sociodemographic and child-related characteristics using proportions and means (and standard deviation).

For aim 1, we summarized the *PTG sum* and *mean score* as well as *the mean domain scores* of bereaved parents with their standard deviation (SD). We also classified the item responses into low (0–1), middle (2–3), and high (4–5) based on previous studies [28].

For aim 2, we compared the PTG scores of bereaved parents to CCS parents using Student *t* test (*PTG sum score*, *PTG mean score*, and *domain mean scores*). To account for differences in sociodemographic and child-related characteristics between bereaved parents and CCS-parents (see Appendix Table S2), we applied inverse probability weighting. These weights were calculated using logistic regression, with those sociodemographic and child-related characteristics as predictor variables, which were different between the groups, and group membership (bereaved parents vs. CCS-parents) as the outcome (for details see Appendix Table S3).

For aim 3, we used univariable linear regression using *PTG sum score* and *domain mean scores* as outcomes to identify factors associated with PTG. We used sociodemographic and child-related characteristics as predictor variables. We included all predictors with *p* value < 0.05 in the multivariable regression. We also explored interaction terms to explore more complex associations.

#### Sensitivity and exploratory analysis

We explored the non-linear relationship of time after death and PTG sum score using splines. A restricted cubic spline with four random knots assignments using mkspline2 package for Stata. We used a model using PTG sum score as the dependent variable and the time after death (4 randomly assigned knots) as independent variable. Plots were done using the smoothing parameters according to the adjustrcspline package for Stata [30]. Details on the spline model parameters and rationale can be found in the Appendix (Appendix Table S4).

### Ethical considerations

Approval from the responsible ethics commission (EKNZ 2021–00906, 4 August 2021 and EKNZ 2015–075; 26 March 2015) was obtained.

## Results

**Fig. 1:**
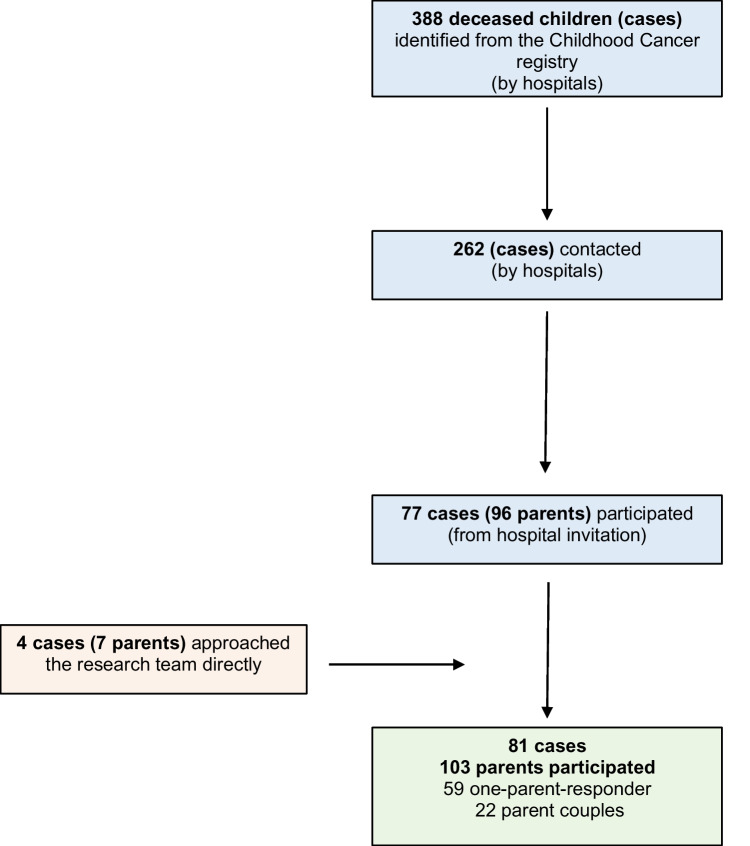
Flow chart of study participants

In the three hospitals, 388 deceased children (cases) were identified by the ChCR. In total, parents of the 262 cases were contacted. After reminder calls, 96 parents of 77 cases completed a questionnaire. Seven additional parents of 4 cases contacted the study team on their own (Fig. 1). Overall, questionnaires from 103 parents of 81 cases were included: 59 one-parent respondents (46 mothers, 13 fathers) and 22 parent couples.

The average age was 53.7 years (SD = 8.3) and the average time after death was 11.3 years (SD = 5.6, Table 1). More children were male (46/81, 56.8%) than female (mean age at the time of death = 9.5 years; SD = 5.9), and most had died from CNS tumors (37/81, 45.7%).

**Table 1:** Sociodemographic profile of bereaved parents

**I. Parents’ characteristics**	***N* = 103**	**%**
**Sex**		
Male	35	34.0%
Female	68	66.0%
**Age group (years)**		
< 50	30	29.7%
50–60	45	44.6%
> 60	26	25.7%
**Migration background**^a^		
Swiss	61	81.3%
With migration background	14	18.7%
**Civil status**		
Single, divorced, widow	18	17.8%
Married	83	82.2%
**Education**		
Compulsory/ Vocational training	71	69.6%
Upper secondary education	12	11.8%
University education	19	18.6%
**Employment/in training**		
Employed	67	65.7%
Unemployed/retired/in training	35	34.3%
**Risk of poverty**^b^		
No risk	37	39.8%
At-risk	56	60.2%
**Practicing religion**		
No	68	70.1%
Yes	29	29.9%
**Time after death**		
< 10 years	43	42.6%
≥ 10 years	58	57.4%
**II. Characteristics of the deceased children**	***N*** **= 81**	**%**
**Sex**		
Male	46	56.8%
Female	35	43.2%
**Child’s age at death**		
< 1 year	3	3.8%
1–5 years	14	17.5%
6–10 years	23	28.7%
> 10 years	40	50%
**Cancer diagnosis**		
Leukemia/lymphoma	22	27.2%
CNS tumor	37	45.7%
Other solid tumors	22	27.1%
**Location of death**		
Hospital/healthcare facilities	35	43.2%
Home	45	56.0%
**Time after the death (year)**	mean = 11.3	SD = 5.6

*CNS* central nervous system, *SD* standard deviation

^a^Migration background is defined as those who has moved to Switzerland, not Swiss citizen, or became citizen at birth of whose parents were not Swiss citizens

^b^Risk-of-poverty was defined as having a monthly household income of < 4500 Swiss Francs (CHF) for single parents and < 6000 CHF for parent-couples. (Mader L, Roser K, Baenziger J, et. al. Household income and risk-of-poverty of parents of long-term childhood cancer survivors. Pediatr Blood Cancer. 2017; 64:e26456. 10.1002/pbc.26456)

### Posttraumatic growth of bereaved parents (Aim 1)

**Fig. 2:**
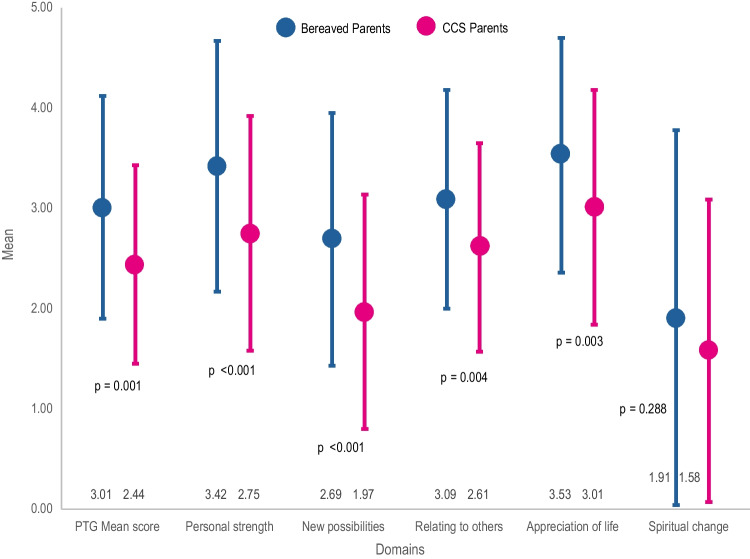
Mean posttraumatic growth domain scores of bereaved parents vs parents of childhood cancer survivors (CCS-parents) based on weighted data

**Fig. 3:**
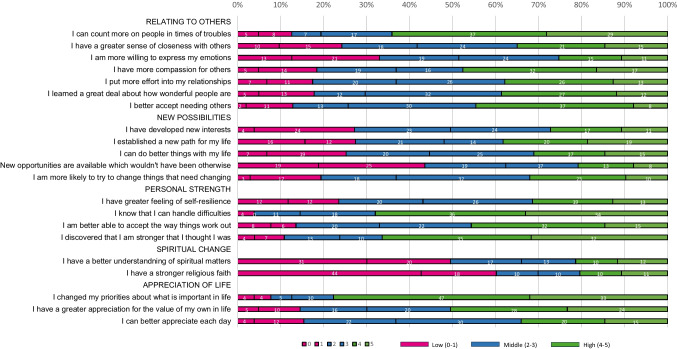
Frequency (%) of bereaved parents by each item categorized by low, middle and high post-traumatic growth scores. Crude numbers are written in each bar

The *PTGI sum score* of bereaved parents had a mean of 63.2 (SD = 23.3, range = 4–105). As for the domains, *appreciation of life* had the highest mean score (3.53, SD = 1.17), followed by *personal strength* (3.42, SD = 1.25), while *spiritual changes* had the lowest (1.91, SD=1.87, Fig. 2). The item “I changed my priorities about what is important in life” was the highest endorsed item (Fig. 3).

### Comparison of bereaved parents to parents whose child survived (Aim 2)

We used data from 584 CCS-parents as a comparison. We found *PTG sum score* to be higher in bereaved parents (mean = 63.2, SD = 23.3) compared with parents whose child survived (51.4, SD = 21.0, *p* = 0.002) (using weighted data). Comparison of *domain mean scores* similarly revealed a higher *appreciation of life* (3.53, SD = 1.17 vs. 3.01, SD = 1.17, *p* = 0.003), *personal strength* (3.42, SD = 1.25 vs. 2.75, SD = 1.17, *p* = 0.001), *relating to others* (3.09, SD = 1.09 vs. 2.61, SD = 1.04 *p*=0.004), and *new possibilities* (2.69, SD = 1.26 vs. 1.97, SD = 1.17 *p* < 0.001) in bereaved parents compared to CCS-parents (Fig. 2). There was no significant difference in *spiritual changes* between the two groups (1.91, SD = 1.87 vs. 1.58, SD = 1.51 *p=*0.288). Crude comparison (non-weighted) can be found in the Appendix, with results aligned with the weighted results (Appendix Table S5).

### Determinants of posttraumatic growth of bereaved parents (Aim 3)

Only time after death and practicing religion were significantly associated with PTG (Table 2). PTG was lower in parents with longer (≥ 10 years) compared to those with shorter time after death. (Coefficient: − 9.0, 95% CI − 17.3, − 0.7 *p*=0.034). Parents practicing religion had higher PTG compared to those not practicing (Coefficient: 10.9, 95% CI 2.1, 19.7, *p*=0.016), which remained significant in the multivariable regression (Coefficient: 10.5, 95% CI 1.6, 19.3, *p*=0.022). The interaction term (time after death × practicing religion) was statistically significant, indicating that parents who had a shorter time after death (< 10 years) and were practicing religion experienced the highest PTG.

**Table 2:** Determinants of post-traumatic growth on bereaved parents

	**Univariable regression**			**Multivariable regression**			**Interaction term**		
	**Coefficient**	**95% confidence interval**	***P* value**	**Coefficient**	**95% confidence interval**	***P* value**	**Coefficient**	**95% confidence interval**	***P* value**
***Sociodemographic characteristics of bereaved parents***									
**Sex (male)**									
Female	4.5	− 4.2, 13.2	0.304		–	–			
**Age (< 50 years)**									
50–60 years	1.9	− 7.6,11.5	0.687	–	–	–			
> 60 years	− 0.4	− 11.2,10.5	0.946	–	–	–			
**Risk of poverty (no)**^**a**^									
At risk	1.5	− 7.3, 10.2	0.739	–	–	–			
**Education category (Compulsory-vocational)**									
Upper secondary	5.0	− 7.6, 17.7	0.429	–	–	–			
University	− 5.8	− 16.2, 4.7	0.274	–	–	–			
**Employment (employed)**									
Unemployed/retired	6.0	− 2.4, 14.8	0.160	–	–	–			
**Civil status (single/divorced)**									
Married	5.6	− 4.9, 16.2	0.292	–	–	–			
**Migration Status (Swiss)**^**b**^									
With migration background	0.8	− 11.8, 13.4	0.896		–	–			
**Practicing religion (no)**									
Yes	**10.9**	**2.1, 19.7**	**0.016**	**10.5**	**1.6, 19.3**	**0.022**	**20.7**	**7.8, 32.9**	**0.002**
**Time after death (< 10 years)**									
≥ 10 years	− **9.0**	− **17.3,** − **0.7**	**0.034**	− **5.9**	− **14.1, 2.3**	**0.159**	**0.01**	− **9.6, 9.8**	**0.985**
***Practicing religion* × *Time after death***^c^							− ***19.1***	− ***36.6,*** − ***1.7***	***0.031***
***Child characteristics***									
**Sex of the child (male)**									
Female	6.8	− 1.4, 14.9	0.105	–	–	–			
**Age at death (< 10 years)**									
≥ 10 years	− 6.0	− 16.2, 4.3	0.251	–	–	–			
**Diagnosis at death (CNS tumor)**									
non-CNS tumor	0.3	− 8.0, 8.6	0.944	–	–	–			
**Location of death (home)**									
Healthcare facility	− 5.1	− 13.4, 3.2	0.225	–	–	–			

*CNS* central nervous system, category in parenthesis indicates the reference group

^a^Risk-of-poverty was defined as having a monthly household income of < 4500 Swiss Francs (CHF) for single parents and < 6000 CHF for parent-couples. (Mader L, Roser K, Baenziger J, et. al.. Household income and risk-of-poverty of parents of long-term childhood cancer survivors. Pediatr Blood Cancer. 2017; 64:e26456. 10.1002/pbc.26456)

^b^Migration background is defined as those who has moved to Switzerland, not Swiss citizen, or became citizen at birth of whose parents were not Swiss citizens

^c^Marginal means of PTG sum scores from a linear regression model with an interaction term (practicing religion × time after death): not practicing, < 10 years = 57.4; not practicing, ≥ 10 years = 57.5; practicing, < 10 years = 77.7; practicing, ≥ 10 years = 58.6

### Sensitivity and exploratory analyses

Sociodemographic and child-related determinants of PTG domains can be found in the Appendix (Appendix Table S6). Time after death is a consistent determinant of PTG, being a predictor for 2 of 5 domains (*appreciation of life* and *new possibilities*). Practicing religion is also a determinant of PTG in 2 of 5 domains (*appreciation of life* and *spiritual change*). Other associations can be seen in the Appendix (Appendix Table S6).

**Fig. 4:**
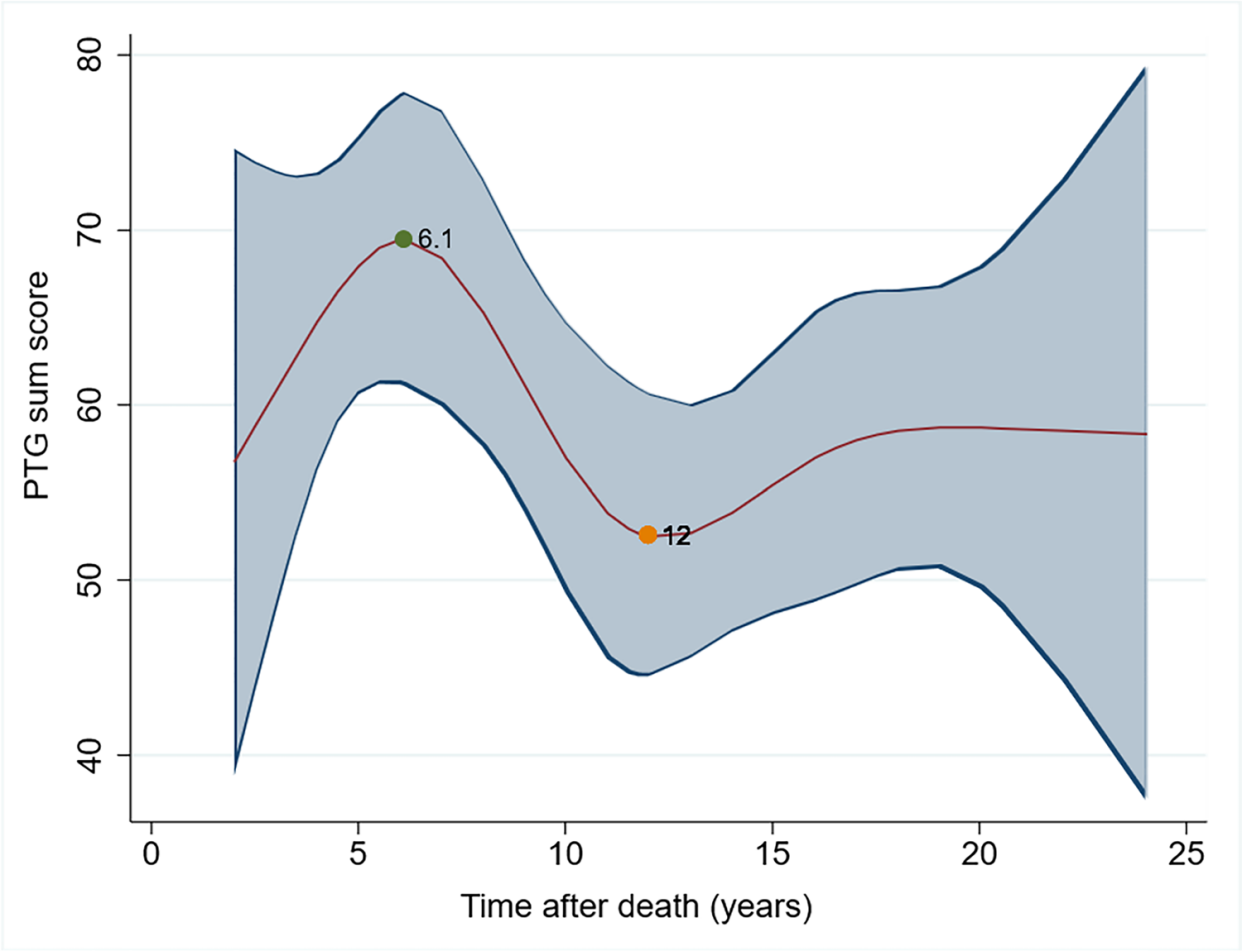
Spline model of PTG sum score and time after death using cross-sectional data (shaded are represents 95% confidence interval, green dot shows maximum inflection point and orange point shows minimum inflection point in terms of time after death in years)

There is a non-linear relationship between time after death and PTG sum score, seen in the spline plot (Fig. 4). PTG sum score at 5 to 8 (peak at 6.2 years) after the death of a child appears to be highest during its trajectory. After that, there is a decreasing PTG sum score as time progresses. After 10 to 15 years, the PTG sum score reaches a plateau.

## Discussion

Parents whose child died from cancer may embrace positive changes in their lives (posttraumatic growth (PTG)), alongside the negative impact of the traumatic event. Our results strongly suggested that bereaved parents’ *PTG* is higher compared to parents whose child survived cancer. These findings are consistent with previous research suggesting that PTG directly corresponds to the severity of the emotional trauma [6]. Furthermore, PTG is higher in the early period (<10 years) following the death and among parents who practiced religion.

The death of a child leads to a profound existential crisis in parents, compelling them to develop coping strategies for self-preservation. Parents often need to reassess their life priorities, refocus their attention, and find new ways to fulfill their parental roles [9, 16]. Grief is recognized as a significant factor promoting PTG. Parents who lost their child may spend more time “ruminating” trying to get a “positive” meaning (meaning-making) from this experience, which might contribute to their higher levels of PTG compared to parents of survivors. However, parents of CCS  also face distinct sources of distress distress, such as worrying about cancer-related late-effects and fear of recurrence [12, 31]. These challenges might explain their higher PTG compared to the general population [20]. Further studies have supported the hypothesis that disease severity might be associated with increased PTG [32, 33].

In contrast to the literature [14], our results indicated no statistically significant difference in total PTG levels between mothers and fathers [13]. This may be potentially related to the lower number of fathers due to lower participation rate. Another reason is that fathers who participated may have better PTG scores than those who did not participate. Nevertheless, although the difference did not reach statistical significance, mothers in our sample exhibited higher PTG score than fathers. This gender difference may be attributed to women’s greater utilization of social support, emotion-focused coping strategies, positive rumination, and seeking professional help, which in turn leads to greater PTG [34, 35].

Another potential explanation may be related to the fact that many mothers are more involved in caregiving throughout the illness [36], have to make significant life changes (e.g., cease employment), and therefore experience greater trauma, resulting in more PTG. It is frequently observed, that bereaved mothers display greater levels of introspection and reflection than other groups. This may manifest itself in various ways, including talking to peers, writing in a journal, or creating artwork, as well as involving meaning-making and keeping bonds [16]. This practice might be associated with better (socially acceptable) coping compared to unexpressed or hidden grief, which is more prevalent among fathers, which can lead to differences in PTG [13, 14]. Further studies on bereaved fathers are required to ascertain how they may experience positive changes in their lives.

We observed higher PTG < 10 years after the child’s death, and lower PTG for those whose child died ≥ 10 years ago. However, we need to be careful when interpreting these findings. A review of studies on PTG in bereaved parents reported that parents who have lost their child < 2 years ago reported lower PTG, suggesting that a recent loss makes it more difficult to identify growth [13]. Immediately after and during the first few years of the child’s death, parents undergo intense emotional and cognitive processing of the circumstances. With time, introspection and reflection may lead to a reappraisal of the experience of losing a child, resulting in increased PTG. Our results showed that particularly parents who practice religion experience higher PTG in the first 10 years after the death of their child. Practicing religion might facilitate receiving various forms of support, from family and friends or support groups, facilitating the coping process. Over time, there can be a decline in social support, the emergence of chronic stressors, the stabilization of daily routines, and emotional fatigue [37]. In addition, response to a traumatic event usually peaks after some period of time and is expected to wane and stabilize as a form of habituation that occurs naturally in most individuals. This aligns with our finding of the dynamic nature of PTG, with initially lower levels of PTG that may rise progressively over time, decline again, and eventually plateau after an extended period. These changes highlight the need for long-term bereavement support and interventions tailored to the shifting emotional and psychological needs of bereaved parents. Our results are aligned with the proposed framework on PTG and Growth After Adversity, which highlights that PTG is an ongoing process, not just a static outcome [7].

Another factor that contributed to higher PTG in bereaved parents was practicing religion. Practicing religion can facilitate the process of meaning-making and provide a sense of purpose [16]. It can foster a sense of belonging to a community and provide a support network for parents [38, 39]. Our finding are consistent with previous studies that showed positive associations between religious practices and higher PTG [14, 40]. However, the spirituality domain of the PTGI showed low mean scores in both CCS and bereaved parents, with no significant group differences. These findings align with broader sociological trends indicating low religious affiliation in Switzerland [41], suggesting that spirituality may not be a relevant PTG domain for both groups.

### Strengths and limitations

There are some limitations that should be considered when interpreting our findings. First, the cross-sectional nature of the study limits the ability to infer causality. We could not determine whether PTG was associated with the child’s death, or with something that happened before or after the event. Although the questionnaire specifically referred to the death of the child, recall and response bias could impact the self-report of PTG. Another key limitation is the low response rate and potential selection bias due to the sensitive nature of the topic. The exclusion of certain parents, due to medical or personal reasons as determined by the hospitals, may have influenced our findings; these parents may experience lower PTG, and our findings may thus overestimate the true difference. Parents may also avoid surveys about their child’s death because of emotional distress, leading to a possible overestimation of PTG in bereaved parents. The lower participation of fathers may have limited the statistical power to detect gender differences. Despite this, the clinical characteristics of the children who died of cancer are similar to the population found in the ChCR, supporting the sample’s representativeness. Finally, the study is limited to the German-speaking regions of Switzerland. Experiences of bereavement may differ in French and Italian-speaking regions, and in migrant populations, potentially limiting the generalizability of findings across Switzerland's diverse linguistic and cultural landscape.

However, our study used data based on the population-based ChCR, which increases the generalizability and robustness of the findings. The relatively large sample size, given the rarity of childhood cancer death, strengthens the statistical power and reliability of the study outcomes. Furthermore, our research is one of the few in Switzerland that specifically focuses on parents whose child died from cancer. A previous assessment in Switzerland included all causes of childhood deaths. Finally, this study uniquely included a large number of bereaved parents who lost their child more than two years ago thereby investigating long-term grief.

### Clinical implications

Similar to the understanding of the potential negative psychological outcomes in bereaved parents, it is important to comprehend positive changes in this population. Identifying factors or interventions that may influence PTG and understanding when it is most likely to occur can help healthcare professionals offer more targeted and effective support tools. Meaning-centered interventions [42], which focus on meaning-making, could be considered in hospital, community settings, or support groups for bereaved parents.

As for future research, cohort studies of parents that include data from before and after bereavement, with long-term follow-up, could provide information on baseline and follow-up status, as well as mitigating factors that might modify psychosocial outcomes. Although this study design might be logistically complex to conduct, it could solve the problem of time-related challenges and disentangle causal relationships.

### Conclusion

Our study strongly suggested that parents who have lost a child to cancer experienced higher levels of PTG than parents whose child survived cancer. Without underestimating the suffering and trauma that parents of survivors may face, this supports previous studies that suggest that PTG may be proportional to the severity of emotional trauma. PTG was found to decrease with time after death and to be higher in parents who practice religion. This knowledge can support healthcare professionals in delivering targeted screening or supportive interventions for certain subgroups of bereaved parents to promote better mental health outcomes.

## Supplementary Information

Below is the link to the electronic supplementary material.

Supplementary file1 (PDF 335 KB)

## Data Availability

Data could be made available upon reasonable request from the corresponding author.

## Abbreviations

CCS: Childhood cancer survivors
CI: Confidence interval
CNS: Central nervous system
ChCR: Swiss Childhood Cancer Registry
PTG: Posttraumatic growth
PTGI: Posttraumatic Growth Inventory
SCCSS: Swiss Childhood Cancer Survivor Study
SD: Standard deviation
