# Supplementary material for: Posttraumatic growth in parents long after their child’s death from cancer—a cross-sectional survey in Switzerland
Source: Support Care Cancer. 2025 Oct 1;33(10):896. doi: 10.1007/s00520-025-09892-x (PMC12488827; doi:10.1007/s00520-025-09892-x)
Supplement: Supplementary file 1 — Supplementary file1 (PDF 335 KB) [file 520_2025_9892_MOESM1_ESM.pdf]

Online Supplement

Posttraumatic growth in parents long after their child's death from cancer - A cross-sectional survey in Switzerland

Eddy Carolina Pedraza 1*, Peter Francis Raguindin1*, Anna Katharina Vokinger 1, Eva De Clercq 1, Manya Jerina Hendriks 1, Eva Maria Tinner 2,3, André Oscar von Bueren 4,5, Katrin Scheinemann 1,6, Eva Bergsträsser 3**, Gisela Michel1**

Author affiliations:

1 Faculty of Health Sciences and Medicine, University of Lucerne, Lucerne, Switzerland

2 Inselspital, University Children's Hospital Bern, Bern, Switzerland

3 Pediatric Palliative Care, University Children's Hospital, Zurich, Switzerland

4 Department of Pediatrics, Obstetrics and Gynecology Division of Pediatric Hematology and Oncology University Hospital of Geneva, Geneva, Switzerland

5 CANSEARCH Research Platform for Pediatric Oncology and Hematology, Faculty of Medicine, Department of Pediatrics, Gynecology and Obstetrics, University of Geneva, Geneva, Switzerland

6 Division of Hematology-Oncology, Children's Hospital of Eastern Switzerland, St Gallen, Switzerland

*Shared first authorship

**Shared last authorship

Correspondence to:

Prof. Dr. Gisela Michel

Faculty of Health Sciences and Medicine

University of Lucerne

Alpenquai 4, 6005 Lucerne, Switzerland

Appendix Table S1: Consensus-Based Checklist for Reporting of Survey Studies (CROSS)

Section/topic	Item	Item description	Reported on page #
**Title and abstract**			
Title and abstract	1a	State the word "survey" along with a commonly used term in title or abstract to introduce the study's design.	1
	1b	Provide an informative summary in the abstract, covering background, objectives, methods, findings/results, interpretation/discussion, and conclusions.	3&5
**Introduction**			
Background	2	Provide a background about the rationale of study, what has been previously done, and why this survey is needed.	5
Purpose/aim	3	Identify specific purposes, aims, goals, or objectives of the study.	5
**Methods**			
Study design	4	Specify the study design in the methods section with a commonly used term (e.g., cross-sectional or longitudinal).	6
Data collection methods	5a	Describe the questionnaire (e.g., number of sections, number of questions, number and names of instruments used).	7
	5b	Describe all questionnaire instruments that were used in the survey to measure particular concepts. Report target population, reported validity and reliability information, scoring/classification procedure, and reference links (if any).	7
	5c	Provide information on pretesting of the questionnaire, if performed (in the article or in an online supplement). Report the method of pretesting, number of times questionnaire was pre-tested, number and demographics of participants used for pretesting, and the level of similarity of demographics between pre-testing participants and sample population.	n.a.
	5d	Questionnaire if possible, should be fully provided (in the article, or as appendices or as an online supplement).	n.a.
Sample characteristics	6a	Describe the study population (i.e., background, locations, eligibility criteria for participant inclusion in survey, exclusion criteria).	6
	6b	Describe the sampling techniques used (e.g., single stage or multistage sampling, simple random sampling, stratified sampling, cluster sampling, convenience sampling). Specify the locations of sample participants whenever clustered sampling was applied.	6
	6c	Provide information on sample size, along with details of sample size calculation.	n.a.
	6d	Describe how representative the sample is of the study population (or target population if possible), particularly for population-based surveys.	6
Survey administration	7a	Provide information on modes of questionnaire administration, including the type and number of contacts, the location where the survey was conducted (e.g., outpatient room or by use of online tools, such as SurveyMonkey).	6
	7b	Provide information of survey's time frame, such as periods of recruitment, exposure, and follow-up days.	6
	7c	Provide information on the entry process:<br>–>For non-web-based surveys, provide approaches to minimize human error in data entry.<br>–>For web-based surveys, provide approaches to prevent "multiple participation" of participants.	6
Study preparation	8	Describe any preparation process before conducting the survey (e.g., interviewers' training process, advertising the survey).	n.a.

Ethical considerations	9a	Provide information on ethical approval for the survey if obtained, including informed consent, institutional review board [IRB] approval, Helsinki declaration, and good clinical practice [GCP] declaration (as appropriate).	8&12
	9b	Provide information about survey anonymity and confidentiality and describe what mechanisms were used to protect unauthorized access.	12
Statistical analysis	10a	Describe statistical methods and analytical approach. Report the statistical software that was used for data analysis.	7-8
	10b	Report any modification of variables used in the analysis, along with reference (if available).	
	10c	Report details about how missing data was handled. Include rate of missing items, missing data mechanism (i.e., missing completely at random [MCAR], missing at random [MAR] or missing not at random [MNAR]) and methods used to deal with missing data (e.g., multiple imputation).	7-8
	10d	State how non-response error was addressed.	8
	10e	For longitudinal surveys, state how loss to follow-up was addressed.	n.a.
	10f	Indicate whether any methods such as weighting of items or propensity scores have been used to adjust for non-representativeness of the sample.	7
	10g	Describe any sensitivity analysis conducted.	8
**Results**			
Respondent characteristics	11a	Report numbers of individuals at each stage of the study. Consider using a flow diagram, if possible.	8
	11b	Provide reasons for non-participation at each stage, if possible.	Fig 1
	11c	Report response rate, present the definition of response rate or the formula used to calculate response rate.	Fig 1
	11d	Provide information to define how unique visitors are determined. Report number of unique visitors along with relevant proportions (e.g., view proportion, participation proportion, completion proportion).	n.a.
Descriptive results	12	Provide characteristics of study participants, as well as information on potential confounders and assessed outcomes.	Tab 1
Main findings	13a	Give unadjusted estimates and, if applicable, confounder-adjusted estimates along with 95% confidence intervals and p-values.	Appendix
	13b	For multivariable analysis, provide information on the model building process, model fit statistics, and model assumptions (as appropriate).	7&9
	13c	Provide details about any sensitivity analysis performed. If there are considerable amount of missing data, report sensitivity analyses comparing the results of complete cases with that of the imputed dataset (if possible).	8&9
**Discussion**			
Limitations	14	Discuss the limitations of the study, considering sources of potential biases and imprecisions, such as non-representativeness of sample, study design, important uncontrolled confounders.	11
Interpretations	15	Give a cautious overall interpretation of results, based on potential biases and imprecisions and suggest areas for future research.	12
Generalizability	16	Discuss the external validity of the results.	11
**Other sections**			

Role of funding source	17	State whether any funding organization has had any roles in the survey's design, implementation, and analysis.	12
Conflict of interest	18	Declare any potential conflict of interest.	12
Acknowledgements	19	Provide names of organizations/persons that are acknowledged along with their contribution to the research.	12

Appendix Table S2. Comparison of sociodemographic and child characteristics of bereaved parents and parents of childhood cancer survivors

	CCS parents	Bereaved parents	P value
	Sociodemographic characteristics		
Age in years (mean, SD)*	62.2 (6.7)	53.7 (8.3)	<0.001
Sex			
Male	244 (41.8%)	35 (34.0%)	0.137
Female	340 (58.2%)	68 (66.0%)	
Migration background			
None	493 (88.7%)	61 (81.3%)	0.068
With	63 (11.3%)	14 (18.7%)	
Civil status			
Single/divorce	80 (14.4%)	18 (17.8%)	0.381
Married	474 (85.6%)	83 (82.2%)	
Education			
Compulsory vocational	366 (66.5%)	71 (69.6%)	0.188
Upper secondary	103 (18.7%)	12 (11.8%)	
University	81 (14.7%)	19 (18.6%)	
Employment			
Employed	331 (58.2%)	67 (65.7%)	0.155
Unemployed	238 (41.8%)	35 (34.3%)	
Risk of poverty*			
No risk of poverty	145 (27.6%)	37 (39.8%)	0.017
At risk of poverty	381 (72.4%)	56 (60.2%)	
Having a religion*			
With religion	494 (84.6%)	71 (69.6%)	<0.001
Without religion	90 (15.4%)	31 (30.4%)	
	Child-related characteristics		
Sex of the child			
Male	319 (54.6%)	56 (54.4%)	0.962
Female	265 (45.4%)	47 (45.6%)	
Cancer diagnosis*			
Leukemia/lymphoma	313 (53.6%)	27 (26.2%)	<0.001
CNS tumor	75 (12.8%)	46 (44.7%)	
Others	196 (33.6%)	30 (29.1%)	
Time after diagnosis*			

<20 years	158 (27.1%)	46 (44.7%)	0.001
20-25 years	146 (25.0%)	23 (22.3%)	
25-30 years	150 (25.7%)	23 (22.3%)	
>30 years	130 (22.3%)	11 (10.7%)	

Abbreviations: CCS, childhood cancer survivors; CNS, central nervous system; SD, standard deviation

*Variable for inverse probability weights modeling

Appendix Table S3. Model selection used for the inverse probability weighting

	Model 1				Model 2			
	Logit	95% CI	p-value	VIF	Logit	95% CI	p-value	VIF
Age	-0.22**	(-0.28,-0.17)	<0.001	7.79	-	-	-	-
Risk of poverty (ref = none)	Ref				Ref			
At risk	-0.70*	(-1.28,-0.12)	0.018	3.40	-0.43	(-0.94, 0.08)	0.096	2.37
Having a religion (ref=with)	Ref				Ref			
Without religion	1.75**	(1.06, 2.43)	<0.001	1.22	1.49**	(0.91, 2.06)	<0.001	1.16
Diagnosis (ref=hematologic)	Ref				Ref			
CNS tumor	1.96**	(1.26, 2.66)	<0.001	1.36	2.06**	(1.44, 2.68)	<0.001	1.24
Others	0.74*	(0.08, 1.41)	0.028	1.61	0.71*	(0.12, 1.31)	0.019	1.45
Time after diagnosis (ref ≤20 years)	Ref				Ref			
20-25 years	-0.16	(-0.89, 0.57)	0.670	1.96	-0.88**	(-1.51, -0.24)	0.007	1.52
25-30 years	1.13**	(0.33, 1.94)	0.006	2.08	-0.49	(-1.11, 0.13)	0.123	1.43
≥30 years	1.75**	(0.67, 2.83)	0.002	2.08	-0.76	(-1.57, 0.06)	0.068	1.43
Intercept	9.99**	(6.94,13.05)	<0.001		-2.08	(-2.71, -1.45)	<0.001	
Pseudo R2		0.328				0.161		

*p <0.05, ** p<0.01

To account for differences in sociodemographic (age, risk of poverty, having a religion) and child characteristics (diagnosis and time after diagnosis), we fitted logistic regression models using the following formula (for model 1):

$$\log\left(\frac{P(\text{bereaved parent})}{1-P(\text{bereaved parent})}\right) = \beta_0 + \beta_1 \cdot \text{age} + \beta_2 \cdot \text{poverty\_risk}$$
$$+\beta_3 \cdot \text{no\_religion} + \beta_4 \cdot \text{CNS} + \beta_5 \cdot \text{other\_malignancy}$$
$$+\beta_6 \cdot \text{yrs20\_25} + \beta_7 \cdot \text{yrs25\_30} + \beta_8 \cdot \text{yrs30plus}$$

Where:

Age = continuous age of the parent

Risk of poverty= binary categorical (reference = no risk)

Having a religion = binary categorical (reference = has religion)

Diagnosis category = 3 levels (reference = leukemia/lymphoma, others = CNS, other malignancies)

Time after diagnosis = 4 levels (reference = ≤ 20 years, others = 20–25, 25–30, ≥ 30 years plus)

We then chose model 2 (model without age due to its high VIF in model 1). Stabilized inverse probability weight was computed using the following formula:

$$\text{swt} = \begin{cases} \dfrac{P(Z=1)}{P(Z=1 \mid X)} & \text{if } Z=1 \\ \\ \dfrac{1-P(Z=1)}{1-P(Z=1 \mid X)} & \text{if } Z=0 \end{cases}$$

Where:

swt = stabilized weight

P(Z=1): as the marginal probability of being a bereaved parent

P(Z=1 | X): as the conditional probability of being a bereaved parent using the logit coefficient from the chosen model

Appendix Table S4. Spline model specifications

Parameters	Rationale
Restricted cubic spline model	Restricted cubic splines were chosen for their flexibility in capturing potential nonlinear associations without overfitting, while maintaining interpretability and smoothness.
4 knots	Exploratory spline modeling begins at 3-5 knots. Four knots is a trade-off providing greater sensitivity to detect potential nonlinear trends in post-traumatic growth over time after death compared to 3 knots, without overfitting the data as might occur with five knots in a sample of this size. Also, likelihood ratio tests were conducted showing 4 knots as the most optimal model

3-knot vs 4-knot model	4-knot vs 5-knot model	3-knot vs 5-knot model
Chi2 = 3.05	Chi2 = 1.25	Chi2 =
p = 0.0806*	p = 0.2628	p = 0.0379*

*Null hypothesis means that simpler the model is sufficient, and that additional parameter does not significantly improve model fit. Lower p value (< 0.05), rejects the null hypothesis, means additional parameter may be a better model fit.

Parameters	Rationale
Random placement of knots	Random knot placement, as opposed to evenly spaced knots, allows the model to adapt more closely to the distribution of PTG sum score, following the exploratory nature of the analysis.
Smoothing parameter	Restricted cubic spline used the randomly assigned knot locations, and adjusted the spline terms by centering them at their mean

Iteration of 3-, 4- and 5- randomly placed knots are shown below:

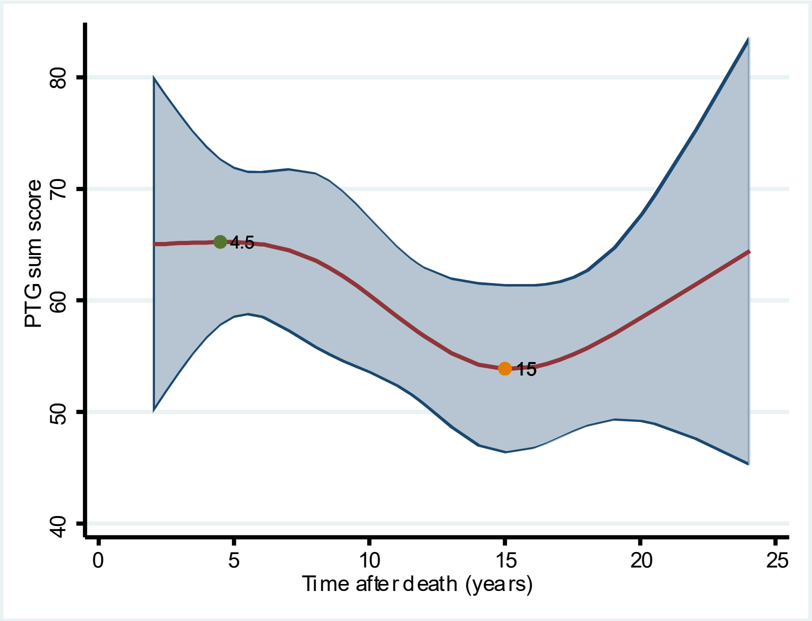

Figure S1. Spline model using 3 randomly placed knots (green dot shows maximum inflection point and orange point shows minimum inflection point in terms of time after death in years, shaded area indicates 95% confidence interval)

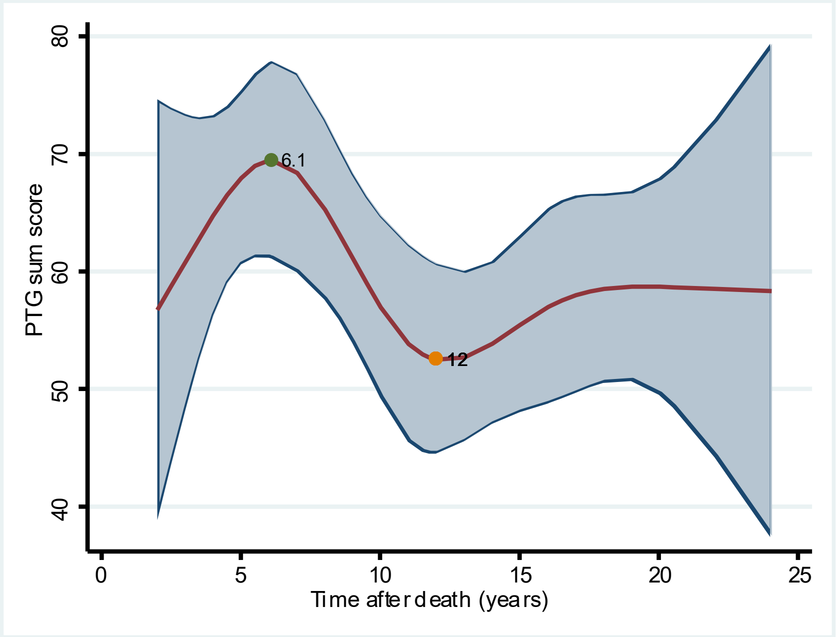

Figures S2. Spline model using 4 randomly placed knots (chosen model, green dot shows maximum inflection point and orange point shows minimum inflection point in terms of time after death in years, shaded area indicates 95% confidence interval)

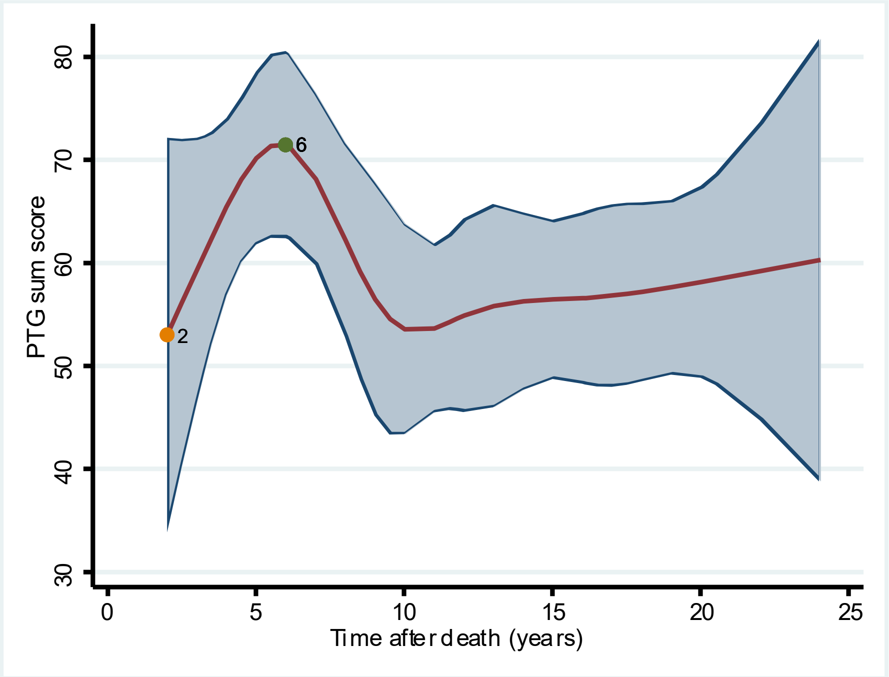

Figure S3. Spline model using 5 randomly placed knots (green dot shows maximum inflection point and orange point shows minimum inflection point in terms of time after death in years, shaded area indicates 95% confidence interval)

Appendix Table S5. Crude comparison (unweighted) of PTG sum score and domain scores between bereaved parents and parents of childhood cancer survivors.

	Parent CCS	Bereaved parents	p value
PTG sum score	51.81 (20.89)	59.79 (21.05)	<0.001
PTG mean score	2.48 (1.00)	2.86 (1.00)	<0.001
Personal strength	2.77 (1.18)	3.27 (1.15)	<0.001
New possibilities	2.01 (1.16)	2.56 (1.18)	<0.001
Relating to others	2.65 (1.05)	2.96 (1.04)	0.005
Appreciation of life	3.05 (1.17)	3.34 (1.14)	0.021
Spiritual change	1.61 (1.52)	1.73 (1.70)	0.483

Abbreviations: CCS, childhood cancer survivor; PTG, post-traumatic growth

Expressed in means and standard deviation.

Appendix Table S6. Univariable linear regression using PTG sum score and domain score as outcomes in bereaved parents

	Univariable Regression														
	**Appreciation of Life**			**New Possibilities**			**Personal Strength**			**Relating to Others**			**Spiritual Change**		
	Coeff.	95% CI	P value	Coeff.	95% CI	P value	Coeff.	95% CI	P value	Coeff.	95% CI	P value	Coeff.	95% CI	P value
**Sociodemographic characteristics of bereaved parents**															
**Age** (<50 years)															
50-60 years	-0.23	-0.74,0.29	0.388	-0.08	-0.63,0.47	0.766	0.26	-0.26,0.78	0.317	0.37	-0.09,0.84	0.114	-0.32	-1.12,0.48	0.425
>60 years	-0.16	-0.75,0.42	0.582	-0.17	-0.80,0.45	0.582	0.01	-0.58,0.60	0.973	0.24	-0.29,0.77	0.372	-0.37	-1.27,0.54	0.427
**Sex** (male)															
Female	0.30	-0.17,0.77	0.209	0.25	-0.24,0.73	0.316	0.26	-0.22,0.74	0.282	0.10	-0.33,0.53	0.648	0.32	-0.38,1.03	0.362
**Risk of poverty** (no)															
**Yes**	0.16	-0.31,0.63	0.512	0.00	-0.49,0.50	0.992	-0.02	-0.51,0.46	0.919	0.01	-0.42,0.44	0.965	0.51	-0.19,1.21	0.153
**Education category** (Compulsory-vocational)															
Upper secondary	0.27	-0.41,0.94	0.435	0.24	-0.48,0.97	0.509	0.26	-0.43,0.95	0.456	0.24	-0.39,0.87	0.447	0.15	-0.90,1.20	0.780
University	-0.31	-0.87,0.25	0.273	-0.22	-0.82,0.38	0.461	-0.42	-0.99,0.14	0.142	-0.10	-0.62,0.42	0.705	-0.67	-1.54,0.20	0.130
**Employment** (employed)															
Unemployed/retired	0.13	-0.33,0.58	0.575	0.31	-0.17,0.79	0.201	0.27	-0.19,0.73	0.255	0.17	-0.25,0.59	0.421	0.89	0.20,1.57	**0.011**
**Civil status** (single/divorced)															
Married	0.36	-0.21,0.92	0.215	0.03	-0.57,0.63	0.924	0.36	-0.22,0.94	0.221	0.17	-0.35,0.69	0.521	0.89	0.03,1.76	**0.042**
Migration Background (Swiss)															
Other countries	-0.08	-0.77,0.60	0.806	0.13	-0.55,0.81	0.702	0.08	-0.61,0.76	0.822	0.06	-0.57,0.70	0.839	-0.17	-1.10,0.77	0.723
**Practicing religion** (no)															
Yes	0.49	-0.01, 0.96	**0.046**	0.39	-0.12,0.91	0.133	0.21	-0.28,0.71	0.391	0.34	-0.10,0.78	0.123	2.10	1.47,2.73	**<0.001**
**Time after death** (<10 years)															
>10 years	-0.51	-0.95,-0.07	**0.025**	-0.53	-0.99,-0.07	**0.024**	-0.30	-0.76,0.16	0.199	-0.38	-0.80,0.03	0.069	-0.46	-1.14,0.21	0.178
**Child-related characteristics**															

**Age at death** **(<10 y)**															
>10 y	-0.33	-0.88,0.23	0.241	-0.19	-0.79,0.41	0.530	-0.50	-1.09,0.09	**0.096**	-0.12	-0.63,0.39	0.639	-0.59	-1.41,0.22	0.150
**Sex of the child** **(male)**															
Female	0.34	-0.10, 0.78	0.131	0.21	-0.25,0.67	0.366	0.54	0.10,0.98	**0.017**	0.21	-0.20,0.62	0.310	0.52	-0.14,1.18	0.123
**Diagnosis at death** **(CNS tumor)**															
Non-CNS tumor	-0.10	-0.55,0.35	0.649	-0.00	-0.47,0.46	0.995	0.01	-0.44,0.47	0.955	-0.03	-0.44,0.38	0.899	0.37	-0.29,1.04	0.270
**Location of death** **(home)**															
Healthcare facility	-0.20	-0.65,0.25	0.380	-0.31	-0.78,0.15	0.184	-0.32	-0.77,0.13	0.166	-0.11	-0.52,0.30	0.593	-0.49	-1.16,0.17	**0.145**

*CI: confidence interval, * Coeff.: Coefficient, *CNS, central nervous system
